# Clinical results of two different three-dimensional plate types for the treatment of mandibular angle fractures: a retrospective analysis

**DOI:** 10.1007/s10006-024-01275-6

**Published:** 2024-06-26

**Authors:** Jelena Pfister, Farah Nur Shazwani, Martin Müller, John-Patrik Burkhard

**Affiliations:** 1 Department of Cranio-Maxillofacial Surgery, Inselspital, Bern University Hospital, University of Bern, Bern, CH-3010 Switzerland; 2 Department of Emergency Medicine, Inselspital, University Hospital Bern, University of Bern, Bern, 3010 Switzerland; 3 Limmat Cleft and Craniofacial Centre, Zurich, CH-8005 Switzerland

**Keywords:** Mandibular angle fracture, Plate design, Three-dimensional plate types, Stress shielding

## Abstract

**Objectives:**

The purpose of this study was to compare two different designs of three-dimensional osteosynthesis plates for their suitability in the treatment of mandibular angle fractures in terms of sufficient fracture healing and concomitant complications.

**Materials and methods:**

Retrospectively a total of 54 patients with 56 mandibular angle fractures were evaluated. Two different types of three-dimensional plates from the Medartis Trilock system were analyzed: (A) Square design plate (☐-plate) with a thickness of 1.0 mm, and (B) triangular-shaped 3D-plate (△-plate) with a thickness of 1.3 mm. Patient demographics, fracture mechanism and intraoperative details were recorded during an average follow-up period of 1 year.

**Results:**

The utilization of △-plates was observed to entail a considerably lengthier surgical time in contrast to ☐-plate systems (*P* = 0.037). The application of △-plate showed a tendency of higher incidence of major complications than ☐-plate (*P* = 0.06), as evidenced by the occurrence of non-union in 2 out of 22 cases, resulting in higher surgical revision rate for △-plate (*P* = 0.027).

**Conclusion:**

Sufficient treatment of mandibular angle fractures is feasible by using 1.0 mm thick, square shaped three-dimensional plate systems. The use of thicker three-dimensional osteosynthesis plates seems to significantly increase the operating time and complication rates, whereby the geometry of the plate seems to have an influence.

**Clinical relevance:**

The plate design could have an impact on treatment outcomes of mandibular angle fractures.

**Trial registration number:**

Not applicable.

## Introduction

Mandibular fractures are among the most common fracture types in the facial skeleton, affecting the jaw angle in up to 36% and the parasymphysis region in about 24% [1]. Men are significantly more frequently affected than women, with rates ranging from 2.9:1 to 5:1 depending on the study population and age, especially in the third and fourth decade of life [1, 2]. The main cause for mandibular fractures is violence, besides falls from great heights, sport and motor vehicle accidents. Notably, in Northern European countries, North America, Australia and New Zealand, people are more prone to interpersonal violence than in third world countries where traffic accidents also frequently lead to this fracture pattern. In short, these vary greatly according to demographic, cultural and socioeconomic factors [2–6]. More specifically for Switzerland, the most frequently cited cause is sports and bicycle-related incidents [4].

The treatment of angular fractures is mostly surgical, with different approaches being used depending on the fracture type. In this regard, the complex anatomy of the mandibular angle with its thin cross-sectional shape, muscular attachments and subsequent masticatory force, as well as the presence of a third molar, must be considered [7, 8]. Various treatment methods have been described for this purpose. Champy’s preferred method of semi-rigid fixation with a load-sharing plate on the oblique ridge is considered the standard method for treating isolated simple mandibular angle fractures. Alternatively, according to the Association of the Study of Internal Fixation (AO), strut plates or three-dimensional plates can be used to treat, ideally, simple mandibular angle fractures. In complicated or comminuted fractures, simplification of the fracture by using several straight plates as well as reconstruction plates is recommended, after which the application of a three-dimensional plates seems to be significantly more difficult with multiple fracture components [9–12].

The use of three-dimensional plates fixed with monocortical screws is considered to be advantageous in simple fractures due to their easier and time-saving handling, as well as their slim design which stabilizes both the tensile and compressive force zones [13, 14]. This potentially implies that, compared to reconstruction and compression plates, a thinner design or reduction in plate cross-section with fewer number of screws per fracture side may be established, even by using monocortical fixation [15]. According to some authors, this may need to be compensated by a higher plate thickness or different plate geometry to achieve sufficient immobilization [16–20]. However, there is still no consensus on the indication and predictability of outcomes with regards to the use of three-dimensional plates for mandibular angle fractures. The aim of this study was to investigate the suitability of two different plate thicknesses and designs for the treatment of mandibular angle fractures in terms of sufficient fracture healing and concomitant complications.

## Material & methods

This retrospective observational study reports a consecutive case series from a single tertiary center. Ethical approval for this study was provided by the Ethical Committee of the Canton Bern, Switzerland (KEKBE 2019-00062) and the need for informed consent was waived.

### Study population

Health-related data of 54 patients with 56 fractures who received open reduction and internal fixation with two different three-dimensional osteosynthesis plates for mandibular angle fracture treatment were evaluated. All patients with simple mandibular angle fractures that were either unilateral or bilateral were included regardless of whether additional facial skull fractures were also present. All forms of dentition were accepted (fully edentulous, partially edentulous, edentulous), but only fully or partially dentulous patients were checked for occlusion. Patients with bone diseases such as osteomyelitis, osteonecrosis after irradiation or treatment with anti-resorptive drugs and those who refused general consent were excluded from this study.

### Data collection and outcomes

**Fig. 1:**
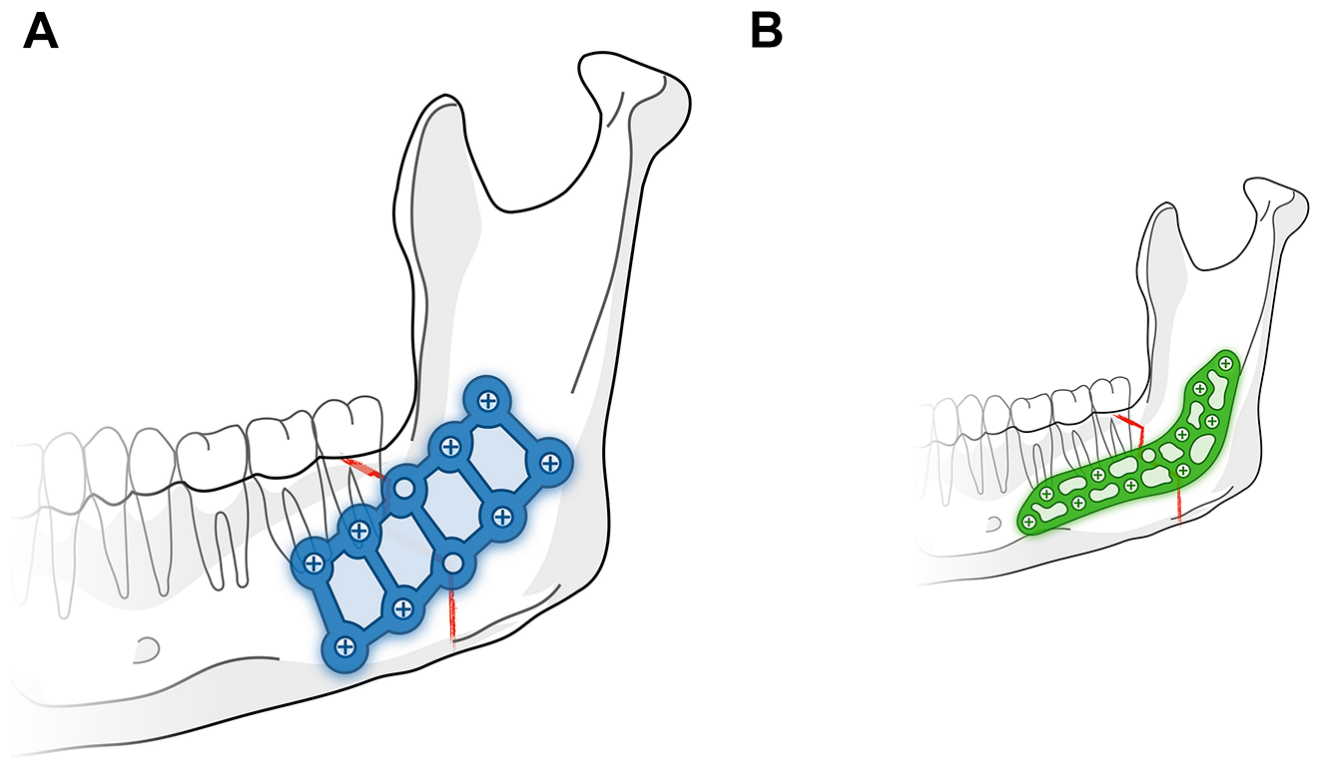
**A** Graphical representation of the square design plate (☐-plate) with a thickness of 1.0 mm. **B** Graphical representation of the triangular-shaped 3D-plate (△-plate) with a thickness of 1.3 mm

Relevant data was extracted from medical records, including paper charts and operation protocols, and stored in the database. Perioperative data collection included age, gender, cause of the fracture, fracture type, plate design, operation time, type and rate of complications and average follow-up period. Two groups were formed (as seen in Fig. 1): 1A) Square design plate (☐-plate) with a thickness of 1.0 mm, and 1B) triangular-shaped 3D-plate (△-plate) with a thickness of 1.3 mm. In addition, the number of screws per fracture side was recorded and categorized as < = 4, or > 4 screws.

The primary outcome was considered to be the achievement of fracture healing defined as radiological evidence of complete bone healing 6 months after treatment. Overall complications were recorded as wound dehiscence, infection, plate fracture, screw loosening or dislocation, sensory disturbance, malposition/non-union, facial paralysis. Major complications required either interventional procedures or revision surgeries. In addition, plate removals were documented, but regardless of whether complications occurred or not.

### Intraoperative and postoperative management

All operations were performed by at least two experienced trauma surgeons from the Department of Cranio-Maxillofacial Surgery, Inselspital Bern, University Hospital and followed the guidelines and standardized house-internal protocol. All surgical procedures performed under general anesthesia with naso-tracheal intubation. Prior to open reduction, dentulous patients received intermaxillary fixation (IMF) with curved splints or IMF screws. Intraoral surgical approach was used in all patients. Preoperatively, co-amoxicillin (1.2 g) or clindamycin (300 mg, penicillin allergy) was administered intravenously and this regimen was continued at least for 24 h postoperatively. The plate for osteosynthesis was selected by the surgeons according to their preferences or based on the fracture line and anatomical conditions. All used plates were from Medartis Trilock system (☐-plate / △-plate, as seen in Fig. 1A and B). Either ☐-plate (1.0 mm) or a △-plate (1.3 mm) was inserted with monocortical screws (with either locking or non-locking screws) with an average length of 5–8 mm. In each case, the IMF screws were either removed intraoperatively or, in the case of condylar fractures with additionally closed fracture reduction, an IMF with elastic rubber bands was inserted for an average of 10 days for additional support. This procedure ended in the outpatient phase when the correct occlusion was achieved (up to 1 week post-operatively). Intraoral wound closure was exclusively performed using absorbable sutures (Vicryl, Ethicon, USA). These sutures naturally dissolve, eliminating the necessity for removal. Patients were instructed to follow a fluid or at least soft diet, to take adequate painkillers and not to undergo excessive physical activity. A postoperative panoramic X-ray was taken after surgery and 6 months postoperatively for quality control. Post-operative follow-up was assessed according to the standardized protocol (1 week, 1 month, 3 months and 6 months after fracture treatment), with special emphasis on occlusion and sensomotoric functions of cranial nerves V and VII.

A fracture was considered stable and healed when there were clinically unrestricted, painless wound conditions with radiological evidence of complete fracture consolidation.

### Statistical analysis

Continuous variables are expressed with median (IQR), p-values obtained by Wilcoxon rank sum test. Categorical variables are shown with number (%) in each category, *P*-values obtained by Chi-squared test. Fisher’s exact test was additionally calculated when appropriate (violation of Chi-squared assumptions). Analyses were performed using the STATA 16.1 (StataCorp, College Station, TX, USA).

## Results

Over a period of 4 years from January 2018 to May 2022, a total of 54 patients with 56 mandibular fractures in two different groups (☐-plate, *n* = 34, 60.7% / △-plate, *n* = 22, 39.3%) were studied and compared for their complications according to the osteosynthesis material used.

**Fig. 2:**
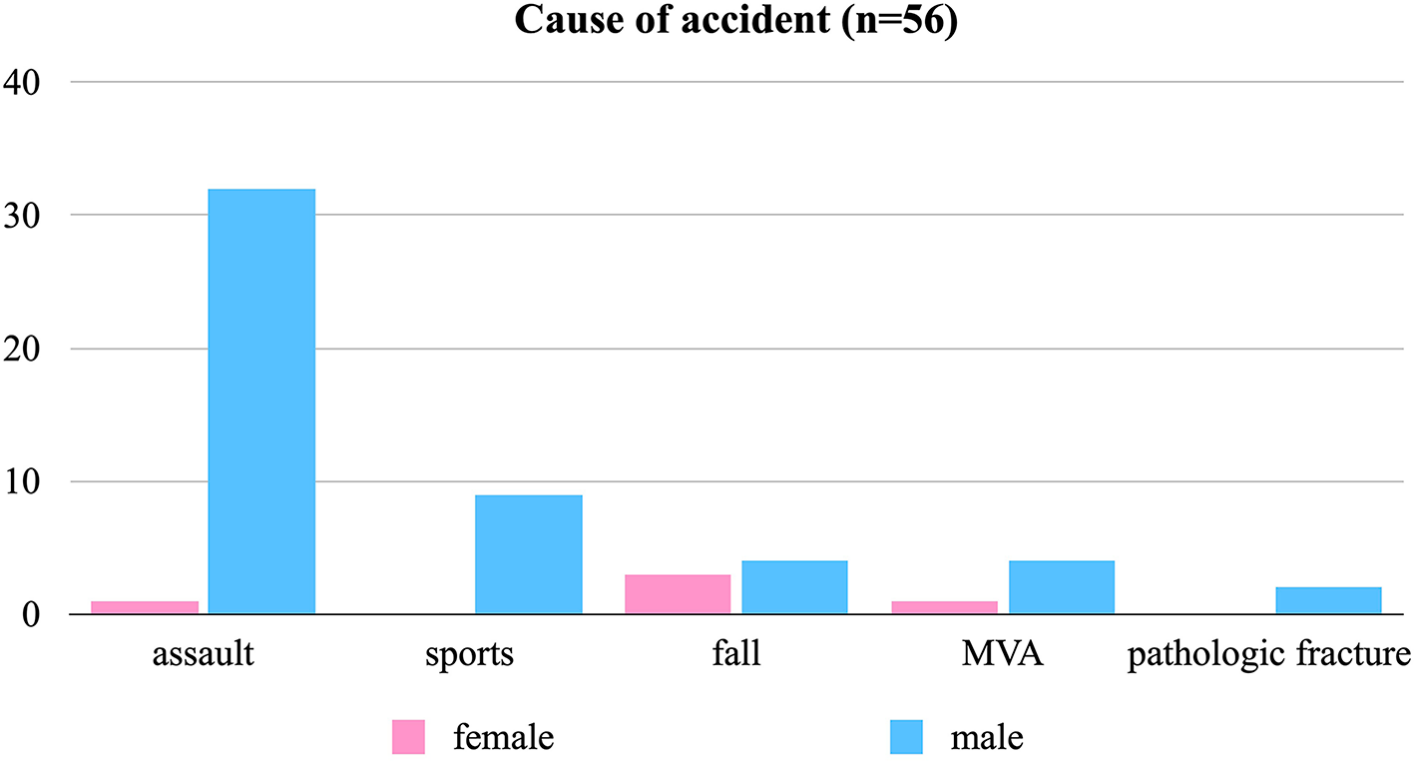
Graphical representation of the cause of the accident by gender

The median age of the patients examined in this study was 22.5 years (ranging from 15 to 89 years old). Among them, 91.1% (*n* = 51) were male and 8.9% (*n* = 5) were female, while 58.9% (*n* = 33) of them were smokers and 17.9% (*n* = 10) were under the influence of alcohol. The most frequent fracture cause was interpersonal violence (*n* = 33, 59%), followed by sport accidents (*n* = 9, 16%), falls (*n* = 7, 13%), pathological fractures (*n* = 2, 4%), as well as bicycle/scooter/e-scooter accidents (*n* = 3, 5%) and car accident (*n* = 2, 4%), see Fig. 2.

Out of the total 56 mandibular angle fractures examined, 42.9% (*n* = 24) were single fractures, while 50% (*n* = 28) were double fractures. Two cases (3.6%) had triple mandibular fractures, and an equal number (3.6%) had combined mandibular and maxillary fractures (*n* = 3). Fractures involving the paramedian or median part of the mandible were observed in 33.9% (*n* = 19) of cases, while the collum mandibulae was involved in 7.1% (*n* = 4). Dislocated fractures were observed in 23.2% (*n* = 13) of patients.

There was no statistically significant difference between the number of screws (≤ 4 and > 4). On average, 4 screws were placed per plate with a mean length of 6 mm in both groups. There was no significant difference in screw length (*P* = 0.954), number of screws per fracture side (*P* = 0.644).

There is significant correlation between type of plate used and the duration of surgery (*P* = 0.037), with an average surgery time of 127.5 min for ☐-plate and 150 min for the △-plate group. The distribution of patients with additional facial injuries/fractures, which required additional surgery time, was balanced in both groups.

Intermaxillary fixation was used in 37 cases (66.1%), which was left in situ for an average of 19 days (range of 12–22 days). In the remaining cases, intermaxillary fixation with elastics was applied only intraoperatively. The group distribution was balanced and not significant between the plates used (*P* = 0.397).

No significant differences but a tendency of higher incidence of complications (*P* = 0.066) were noted between the two plate types, with twice the number of cases in the △-plate group requiring significantly more revision surgery (*P* = 0.027). Regarding the ☐-plate group, the cases classified as complications included a partial plate fracture with complete consolidation of the fracture in one patient, while the other two cases involved wound dehiscence and local infection with transient sensory disturbance. These could be treated by topical drug application and systemic antibiotic therapy, respectively. Nevertheless, none of these cases required revision surgery.

Six out of nine cases (27.3%) treated with △-plate exhibited complications. Non-union was observed in two cases. The remaining four cases experienced wound dehiscence, partial plate fracture, postoperative sensitivity disorder, or infection. Three of these cases required revision surgeries, with two receiving re-osteosynthesis (reconstruction plate) with fracture cleansing and one receiving abscess drainage. There was no statistically significant difference in the overall duration of hospital stay between the two groups (*P* = 0.581). The median duration of antibiotic administration was 5 days for △-plate and utmost one day for ☐-plate, with no statistically significant difference between the two plate types (*P* = 0.235). Table 1 provides a summary of all values.

**Table 1:** Baseline clinical and demographic parameters with surgery related parameters and complications

Variables	All	Total	(*n* = 56)	△-plate	(*n* = 22)	☐-plate	(*n* = 34)	*P*-value	*P*-value (exact)
**Demographic parameters**									
Age [years]	56	22.5	[19; 32]	24.5	[19; 38]	21	[19; 27]	0.210	
Gender	56							0.973	1.000
Male		51	[91.1]	20	[90.9]	31	[91.2]		
Female		5	[8.9]	2	[9.1]	3	[8.8]		
**Fracture related parameters**									
Dislocation	56	13	[23.2]	6	[27.3]	7	[20.6]	0.563	
**Surgery related parameters**									
Time to Surgery [min]	56	2	[1; 4.5]	2	[1; 5]	2	[1; 3]	0.388	
Duration OP [min]	56	148	[105.5; 180]	150	[120; 210]	127.5	[100; 160]	0.037*	
No. of screws	56	4	[4; 5.5]	4	[4; 5]	4	[4; 8]	0.644	
> 4 screws used	56	18	[32.1]	7	[31.8]	11	[32.4]	0.967	
Plate shortened	56	4	[7.1]	1	[4.5]	3	[8.8]	0.544	
Screw length	56	6	[6; 7]	6	[6; 7]	6	[6; 7]	0.954	
IMF received	56	37	[66.1]	16	[72.7]	21	[61.8]	0.397	
IMF Duration [days]	33	19	[12; 26]	16.5	[12; 21]	20	[12; 30]	0.250	
Length of hosp. [days]	56	3	[2; 3.5]	3	[2; 4]	2	[2; 3]	0.581	
Antibiotic duration [days]	56	1	[1; 6]	5.5	[1; 7]	1	[1; 5]	0.235	
**Complication related parameters**									
Complication (major)	56	9	[16.1]	6	[27.3]	3	[8.8]	0.066	
Wound dehiscence	56	3	[5.4]	2	[9.1]	1	[2.9]	0.318	
Non-union	56	2	[3.6]	2	[9.1]	0	[0.0]	0.073	
Infection	56	3	[5.4]	2	[9.1]	1	[2.9]	0.318	
Plate break	56	2	[3.6]	1	[4.5]	1	[2.9]	0.752	
Screw dislocation	56	1	[1.8]	1	[4.5]	0	[0.0]	0.210	
Sensitivity disorder (persistent)	56	3	[5.4]	1	[4.5]	2	[5.9]	0.314	0.329
Facial nerve weakness	56	0	[0.0]	0	[0.0]	0	[0.0]	-	
Malocclusion	56	0	[0.0]	0	[0.0]	0	[0.0]	-	
Trismus	56	0	[0.0]	0	[0.0]	0	[0.0]	-	

## Discussion

This retrospective study compares the clinical outcome of two three-dimensional osteosynthesis plates of different plate geometry and thickness.

Three-dimensional plates provide the advantage of stabilizing fracture sides in a rigid manner against forces in three dimensions, including shear, bending, and torsional forces [21]. The ease of handling three-dimensional plates and their impact on surgery time remains unclear [15, 22, 23], as it may depend on various factors, such as surgeon experience, fracture pattern, plate thickness, and geometry. This study shows that plates with different designs, such as the △-plate, may be more difficult to handle, what is reflected in the longer operation times, as the bending of these plates is much more demanding. This seems to be one reason why there is a tendency in the literature to use three-dimensional plates in fractures with minimal or no displacement. Nevertheless, their use is independent of location and associated with reliable outcome prediction [24]. However, some studies have suggested the need for caution when using three-dimensional plates in mandibular angle fractures [15, 25].

Mandibular angle fractures place special demands on adequate fracture treatment due to the high tensile forces of the attaching musculature [26]. Mobile fragments, inadequate and unstable fracture reduction or malcompliance of the patient itself can be the reason for postoperative infections, non-unions and treatment failures. Therefore, the stability of the plates are seen to be crucial to keep these problems low, especially in the case of condylar head fractures [27–29]. However, the use of open reduction and stable internal fixation in treating angle fractures is still associated with a high complication rates, where three-dimensional plating systems are accounted for about 15–53% [25, 30–32].

The relatively low incidence of wound dehiscence and infection, reported in the current literature similar to the findings of this study, can be attributed to the use of careful surgical techniques and adherence to established treatment protocols, including the use of antibiotics [33]. However, some authors argue that postoperative wound infections are principally caused by fracture mobility and should be addressed by increasing fracture immobilization [15]. Even indicating an inverse relationship between the stiffness of the fixation and the occurrence of complications [34]. In comparative studies, three-dimensional plates exhibited superior performance when related to alternative methods of fixation such as a 2.0 mm plate, two 2.0 mm plates, a reconstruction plate, or MMF. This is likely due to the stability provided in three dimensions and resistance to torque and malleability. The success rate of three-dimensional plates was over 80%, except in three comparative studies [13, 22, 35].

However, it should be noted that depending on the type and localization of the fracture, the requirements for the plates and their design change [1, 6, 36].

The success of an osteosynthesis plate lies primarily in an assured fracture healing, which could not be achieved in two cases in the present study. Also in the comparative literature, non-unions were found with three-dimensional plates [13]. Of course, the surgeon’s inexperience or the patient’s characteristics, such as existing diseases or patients’ non-compliance, inadequate fracture reduction and instability can also be responsible for a non-union. Since adequate blood supply is essential for fracture healing, depending on the size of the plate to be inserted, more extensive deperiosteation has to be done, which impairs blood supply [37]. Whether this plays an additional role requires further investigation. In the two cases in this study, however, this seems unlikely, which is why another, less recognized cause must be considered, that of stress shielding.

Stress shielding is a phenomenon that occurs when the mechanical load on a bone decrease as a result of the insertion of a stiff implant. This effect has been observed in the use of rigid internal fixation plates on mandibular bone grafts. When the bone is not subjected to the normal stress and strain patterns, it can result in a reduction of bone density and weakening of the bony structure over time. This can occur due to the mechanical properties of the implant causing it to bear more of the load than the bone itself, leading to a reduction in the natural bone remodeling process. Potentially resulting in implant loosening or failure and may require revision surgery [38].

Based on the findings, plate fractures in this study only occurred after the fracture had completely healed. These plates are possibly too rigid, i.e. a combination of plate size, geometry and thickness, will suppress transmission of micromovements that could stimulate the bone. In this sense, the △-plate may have too rigid fixation in the mandibular angle region if it is too large, after which a reduction in plate size could be helpful. This study is restricted by the limited number of patients. Nevertheless, both plate designs seem to be feasible for the treatment of mandibular angle fractures, whereby the ☐-plate performed better. Further studies should address this question. In conclusion, a sufficient treatment of mandibular angle fractures is feasible by using 1.0 mm square-designed plate systems. The employment of thicker three-dimensional, triangular-designed osteosynthesis plates appears to substantially prolong the surgical duration and elevate the risk of complications.

## Data Availability

The study data cannot be shared, as this was not permitted in the application to the Ethics Committee, as it concerns sensitive patient data.
